# The impact of preoperative nutritional screening, ERAS protocol, and mini-invasive surgery in surgical oncology: A multi-institutional SEM analysis of patients with digestive cancer

**DOI:** 10.3389/fnut.2023.1041153

**Published:** 2023-03-16

**Authors:** Laura Lorenzon, Riccardo Caccialanza, Valentina Casalone, Gloria Santoro, Paolo Delrio, Francesco Izzo, Marco Tonello, Maria Cristina Mele, Carmelo Pozzo, Paolo Pedrazzoli, Andrea Pietrabissa, Piero Fenu, Alfredo Mellano, Elisabetta Fenocchio, Antonio Avallone, Francesca Bergamo, Maria Teresa Nardi, Roberto Persiani, Alberto Biondi, Flavio Tirelli, Annamaria Agnes, Renato Ferraris, Virginia Quarà, Michela Milanesio, Dario Ribero, Marilena Rinaldi, Paola D'Elia, Maurizio Rho, Carola Cenzi, Domenico D'Ugo

**Affiliations:** ^1^Fondazione Policlinico Universitario Agostino Gemelli IRCCS, Catholic University, Rome, Italy; ^2^Fondazione IRCCS Policlinico San Matteo, Pavia, Italy; ^3^Candiolo Cancer Institute–IRCCS, Turin, Italy; ^4^Istituto Nazionale per lo Studio e la Cura dei Tumori Fondazione Giovanni Pascale IRCCS, Naples, Italy; ^5^Veneto Institute of Oncology IOV-IRCCS, Padua, Italy

**Keywords:** structural equation modeling (SEM), nutritional screening, surgical oncology, mini-invasive surgery, digestive cancers

## Abstract

**Background:**

Mini-invasive surgery (MIS), ERAS, and preoperative nutritional screening are currently used to reduce complications and the length of hospital stay (LOS); however, inter-variable correlations have seldom been explored. This research aimed to define inter-variable correlations in a large series of patients with gastrointestinal cancer and their impact on outcomes.

**Methods:**

Patients with consecutive cancer who underwent radical gastrointestinal surgery between 2019 and 2020 were analyzed. Age, BMI, comorbidities, ERAS, nutritional screening, and MIS were evaluated to determine their impact on 30-day complications and LOS. Inter-variable correlations were measured, and a latent variable was computed to define the patients' *performance status* using nutritional screening and comorbidity. Analyses were conducted using structural equation modeling (SEM).

**Results:**

Of the 1,968 eligible patients, 1,648 were analyzed. Univariable analyses documented the benefit of nutritional screening for LOS and MIS and ERAS (≥7 items) for LOS and complications; conversely, being male and comorbidities correlated with complications, while increased age and BMI correlated with worse outcomes. SEM analysis revealed that (a) the latent variable is explained by the use of nutritional screening (p0·004); (b) the variables were correlated (age–comorbidity, ERAS–MIS, and ERAS–nutritional screening, *p* < 0·001); and (c) their impact on the outcomes was based on direct effects (complications: sex, p0·001), indirect effects (LOS: MIS-ERAS-nutritional screening, *p* < 0·001; complications: MIS-ERAS, p0·001), and regression-based effects (LOS: ERAS, MIS, *p* < 0·001, nutritional screening, p0·021; complications: ERAS, MIS, *p* < 0·001, sex, p0·001). Finally, LOS and complications were correlated (*p* < 0·001).

**Conclusion:**

Enhanced recovery after surgery (ERAS), MIS, and nutritional screening are beneficial in surgical oncology; however, the inter-variable correlation is reliable, underlying the importance of the multidisciplinary approach.

## Introduction

Enhanced recovery after surgery (ERAS) is a multidisciplinary pathway established to improve surgical patient care. The approach is based on the collaboration and synergy of different physicians, including surgeons, anesthesiologists, gastroenterologists, and clinical nutritionists, and aims to reduce the response to surgical stress, optimize patients' physiological function, and facilitate recovery.

Since the initial experiences in colorectal surgery in the 90's (1), its application increased and was extended to different subspecialties, as several clinical studies documented a reduction of complications and recovery time by 30–50% (2).

Although it is composed of several procedures, the protocol is now perceived as a way of working, an evolving *modus operandi*, rather than a collection of procedures that includes a preoperative phase (pre-habilitation and optimization of patients' correctable deficits, such as anemia or malnutrition), an intra-operative phase (surgical and anesthesiologic optimization), and a postoperative period (enhanced hospital recovery and post-discharge phase).

Indeed, the ERAS pathway is structured on several articulated measures linked to the common ground of improving patients' physiology before the surgical intervention (stress event); these measures could be divided into “general” and “procedure-specific” measures, which balance ERAS measures with the specific risks associated with the different gastrointestinal (GI) procedures (3).

In particular, the pre-habilitation phase is common in all cancer subspecialties, and preoperative nutritional screening is one of its pillars. Malnutrition in patients with cancer undergoing surgery has been associated with a poor prognosis, higher costs, longer length of hospital stay (LOS), and increased risk of postoperative complications (4). As expected, malnutrition is very common in GI surgery and affects 15–30% of patients with colorectal cancer (CRC), upper gastrointestinal (UGI)/gastroesophageal cancer, and pancreatic cancer (5–9). Alarmingly, these figures are greater in elderly patients (>70 years old), in patients with retroperitoneal sarcomas candidate for multi-visceral resection, and in metastatic patients undergoing cytoreductive surgery and hyperthermic intraperitoneal chemotherapy for peritoneal and surface malignancies (PSM) (10–12).

However, despite the several pieces of evidence available that support the use of preoperative nutritional screening tools (13), their application in clinical practice is worldwide neglected (14). The sub-optimal translation in clinical practice of what should be nowadays considered as “best practice” also affects the use of mini-invasive surgery (MIS) (15), which is another landmark of ERAS (4).

Although the application of ERAS, nutritional screening, and MIS was documented as cost-effective in CRC, particularly with respect to LOS (16), the relations between these features and their direct or indirect effects on the outcomes were not explored in CRC or in other GI malignancies.

Indeed, weighing the impact of all these interrelated variables is challenging, and in this context, the use of structural equation modeling (SEM) could contribute to the analysis and interpretation of results. To date, clinical studies have focused on the relation of single items with clinical outcomes or adverse events using standard multivariable analyses but have not explored the relationship among variables (including the aforementioned). This interrelation and the “collinearity” of variables are usually understudied. However, the clinical variables used in medicine often present several degrees of collinearity (for example, age and the presence of comorbidity), and an SEM approach has the potential to overcome this issue.

Structural equation modeling (SEM) analysis is a set of statistical techniques that combines different regression models used to describe the relationship among observed variables and their linear causal relationships while simultaneously accounting for measurement error. It also allows the analysis of latent factors, not directly observed or measured but defined by other observed features. Latent variables are used to translate the fact that several observed and measurable variables are imperfect measurements of a single underlying concept. In other fields, examples of latent variables include quality of life, business confidence, morale, happiness, and conservatism. All these are variables that cannot be measured directly. SEM has been extensively used in economics, sociology, and behavioral science, but its current application in clinical medicine is scant, probably because of technical difficulties (17).

Thus, this research aimed to explore the impact of MIS, ERAS, and preoperative nutritional screening on 30-day complications and LOS in a large multi-institutional dataset of patients with GI cancer undergoing surgery. The primary aim was to define the inter-variable correlations and the impact of direct (single variable) and indirect (multiple correlated variables) effects on the outcomes.

## Methods

### Patients and setting

Patients who underwent elective surgery with curative intent upfront and after neoadjuvant therapy for CRC, UGI, and hepatobiliary–pancreatic (HPB) malignancies, including metastatic and patients with PSM candidates for cytoreductive surgery with/without intraoperative chemotherapy or other malignancies requiring a surgical resection of the gut in 2019 and 2020 at five Italian research hospitals (Fondazione Policlinico Universitario Agostino Gemelli-Rome, Fondazione Policlinico San Matteo–Pavia, Istituto Nazionale Tumori Fondazione G. Pascale-Naples, Istituto Oncologico Veneto–Padua, Istituto di Candiolo Fondazione del Piemonte per l'Oncologia–Candiolo, Turin) were reviewed and analyzed. Patients were excluded if they were <18 years of age, had missing data, or had undergone palliative or urgent/emergency procedures. The hospital setting, nutritional evaluation, and use of nutritional screening tools in participating centers were portrayed using a quality analysis, as described before (14) and presented in the Supplementary File. The research protocol was submitted to the Italian Ministry of Health and financed as part of the network projects of Alleanza Contro il Cancro (the National Oncology Network founded in 2002 and participated by 28 institutes for comprehensive cancer care and research), but it was not pre-registered.

### Clinical records

For data collection, a database was designed adhering to the STROBE statement for collection, interpretation, and divulgation of results (18). Clinical variables were established on the basis of the preliminary qualitative analysis. All clinical records were recorded by recruiting centers, de-identified, and then pooled anonymously in a common database by the PI using consecutive numbers.

Demographic data (age at the time of the procedure and sex), presence of comorbidities (defined as Charlson index >3), tumor site, nutritional data (BMI and preoperative nutritional screening evaluation independently from the tool), surgical variables (year and type of procedure and use of MIS), and adherence to the ERAS protocol (defined as the application of at least seven items) (19) were collected.

### Outcomes of interest

The outcomes of interest were postoperative LOS (measured in days) and 30-day postoperative complications, regardless of their severity.

### Statistical analysis

Continuous variables were reported as means and standard deviations (SD) or median and interquartile ranges (IQRs), whereas categorical variables were reported as frequencies and percentages. Statistical analyses followed a three-step approach: first, univariable analyses were performed, and second, correlation and partial correlation were identified. These steps allowed the identification of possible direct effects (first step), the degrees of collinearity among variables, and those presenting the strongest uniqueness criteria (absence of collinearity) to define indirect effects and the latent variable (see below). Third, the final analysis was conducted.

On this basis, and as a first step, quantitative variables were analyzed using parametric *t*-tests and non-parametric Mann–Whitney tests, according to the distribution of variables, whereas qualitative χ2 tests were performed for categorical variables. Two-tailed univariable analyses were performed for the two outcomes of interest, and a *p*-value of 0·05 was considered statistically significant. As a second step, correlations between the qualitative (binary and ordinal) and quantitative variables were tested. Qualitative variables were tested using polychoric and polyserial correlations, whereas Pearson's correlation was used to test quantitative variables (*polycor* package, R software). An exploratory factor analysis (EFA) was then performed, including the Kaiser-Meyer-Olkin test and Bartlett's test for sphericity, to measure the sampling adequacy and homogeneity of variance (*parameters* package, R software). Correlations were obtained and defined as strong if ranging between 1·00 and 0.80, medium if between 0.79 and 0.50, moderate if between 0.49 and 0.20, or weak if <0.19. Confirmatory factor analysis (CFA) was conducted, and partial correlations were evaluated with the exclusion of exogenous variables/outcomes: LOS and postoperative complications. These partial correlations and results of the factorial analysis were used to define a latent variable that could summarize the patient's *performance status* without any collinearity. Finally, as a third step, the latter and all the endogenous variables were computed in an SEM analysis including multivariable regression, inter-variable correlations, and the measure of direct (of a single variable) and indirect effects (of multiple correlated variables) on the outcomes. As specified earlier, the variables computed for SEM analysis were defined by the results of the univariable tests and the partial correlations, as shown in the Supplementary File. A SEM analysis was performed using the *lavaan* package of the R software (https://cran.r-project.org/) (20, 21).

## Results

### Study population

**Figure 1:**
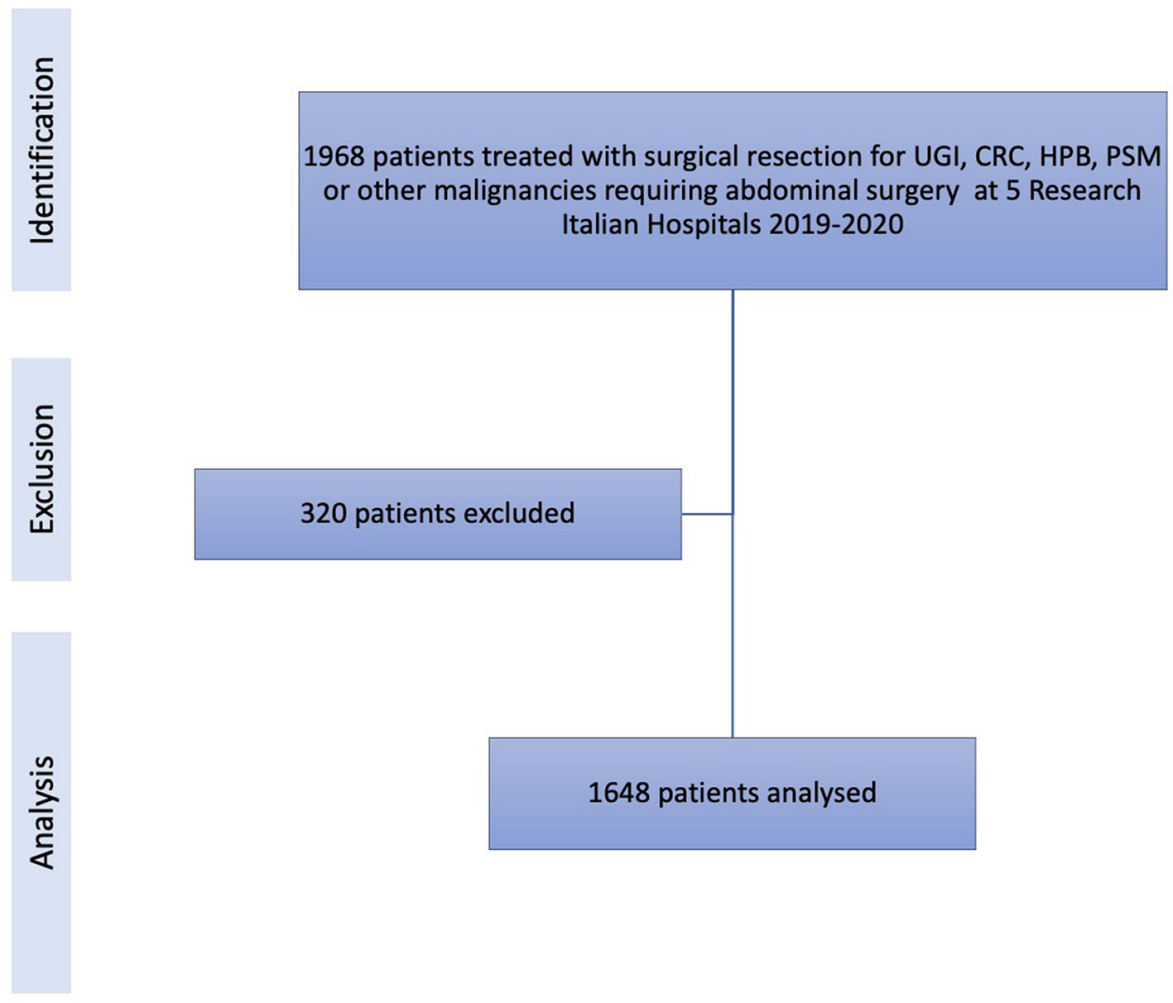
STROBE flowchart of the study population showing identification of the cohort, exclusion of patients, and the final selection of patients analyzed.

Of the 1,968 patients registered between 2019 and 2020, 320 were excluded due to missing data; thus, 1,648 patients (1,041 CRC, 177 UGI, 125 HPB, 268 metastatic patients with or without PSM, and 37 patients with other malignancies requiring abdominal surgery) were included in the data analysis (Figure 1).

The clinical settings and practices of the participating institutions are presented in Supplementary Figure 1. Overall, the key elements of nutritional evaluation were routinely collected in all institutions and for each subspecialty, even though few discrepancies were noted for HPB and CRC surgeries (Supplementary Figure 2).

Regarding the cohort analyzed, the mean age of the patients was 65.9 years, and the mean BMI was 25.8. Almost half of the patients were treated with MIS, and 71.8% of patients had at least one comorbidity. Of note, 85.5% of the patients underwent nutritional screening, and 23.9% were treated with perioperative nutritional support (before/after surgical resection). The mean LOS was 8.5 days, and 26.7% of the patients presented with at least one postoperative complication (Supplementary Table 1). A breakthrough of these clinical features in different cancer types is presented in Supplementary Table 2. Overall, when comparing subgroups, there was no significant difference regarding age (CRC mean age: 67.2 years, SD 12.1, range 19–91 years; UGI mean age: 68.1 years, SD 12.1, range 34–96 years; HPB: mean age 68.5 years, SD 10.5, range 20–85 years; *t*-test: CRC vs. UGI, p 0.34; CRC vs. HPB, p 0.22; UGI vs. HPB, p 0.08), but there was a prevalence of male in UGI and HPB patients comparing CRC (respectively, 62.1 and 61.6 vs. 50.9%, chi-square *p* 0.003), and probably as expected, we documented a difference in BMI comparing CRC/HPB and UGI patients (CRC mean BMI: 26.0, SD 4.3, range 15.6–53.8; UGI mean BMI: 24.9, SD 4.2, range 14.9–38.6; HPB: mean BMI 25.9, SD 3.9, range 18.5–40.1; *t*-test: CRC vs. UGI, *p* 0.002; CRC vs. HPB, *p* 0.85; UGI vs. HPB, *p* 0.04).

Statistical analyses showed that the use of MIS, the practice of preoperative nutritional screening, and adherence to the ERAS protocol were inversely correlated with LOS; conversely, increased age and BMI were directly correlated with increased LOS. Being male, presence of comorbidity, increased age, and BMI correlated with postoperative complications; opposite findings were reported in univariable analyses with respect to the use of MIS and ERAS practices (Table 1). The vast majority of these findings were confirmed in the three most representative subgroups of the cohort (CRC, UGI, and HPB), given, however, the absence of ERAS practice in UGI patients (Supplementary Tables 3–5).

**Table 1:** Univariable analyses in the cohort.

	**LOS ≤ 6 days**	**LOS ≥7 days**	***p*-value**	**30-Days Complications No**	**30-Days Complications Yes**	***p*-value**
	***n* patients (%)**	***n* patients (%)**		***n* patients (%)**	***n* patients (%)**	
**Mini-invasive surgery**						
Yes	599 (65.6%)	219 (29.8%)	**<0.01** **^§^**	655 (54.2%)	163 (37.1%)	**0.01** **^§^**
No	314 (34.4%)	516 (70.2%)		553 (45.8%)	277 (62.9%)	
**Sex**						
F	454 (49.7%)	349 (47.5%)	0.39^§^	621 (51.4%)	182 (41.4%)	**0.01** **^§^**
M	459 (50.3%)	386 (52.5%)		587 (48.6%)	258 (58.6%)	
**Nutritional screening**						
Yes	841 (92.1%)	568 (77.3%)	**<0.01** **^§^**	1,023 (84.7%)	386 (87.7%)	0.14^§^
No	72 (7.9%)	167 (26.7%)		185 (15.3%)	54 (12.3%)	
**Co-morbidity**						
Yes	644 (70.5%)	539 (73.3%)	0.23^§^	849 (70.3%)	334 (75.9%)	**0.028** **^§^**
No	269 (29.5%)	196 (26.7%)		359 (29.7%)	106 (24.1%)	
**ERAS (**≥**7 items applied)**						
Yes	635 (69.6%)	216 (29.4%)	**<0.01** **^§^**	681 (56.4%)	170 (38.6%)	**0.01** **^§^**
No	278 (30.4%)	519 (70.6%)		527 (43.6%)	270 (61.4%)	
**Age (years)**						
Median (range)	67.0 (19.0–89.0)	68.0 (20.0–96.0)	**≤0.01** **^§§§^**	67.0 (19.0–96.0)	68.1 (19.0–92.0)	**≤0.01** **^§§§^**
**BMI**						
Median (range)	26.0 (16.0–54.0)	25.0 (15.0–47.0)	**≤0.01** **^§§§^**	25.4 (15.0– 54.0)	25.3 (15.0– 46.0)	**≤0.01** **^§§§^**

LOS, Length of post-operative hospital stay (above and below the median value); 30-Days Complications, 30-days post-operative complications.

§ Chi square test;

§§§ Mann-Whitney test. Bold is for statistical significance.

### Variables' correlation

**Figure 2:**
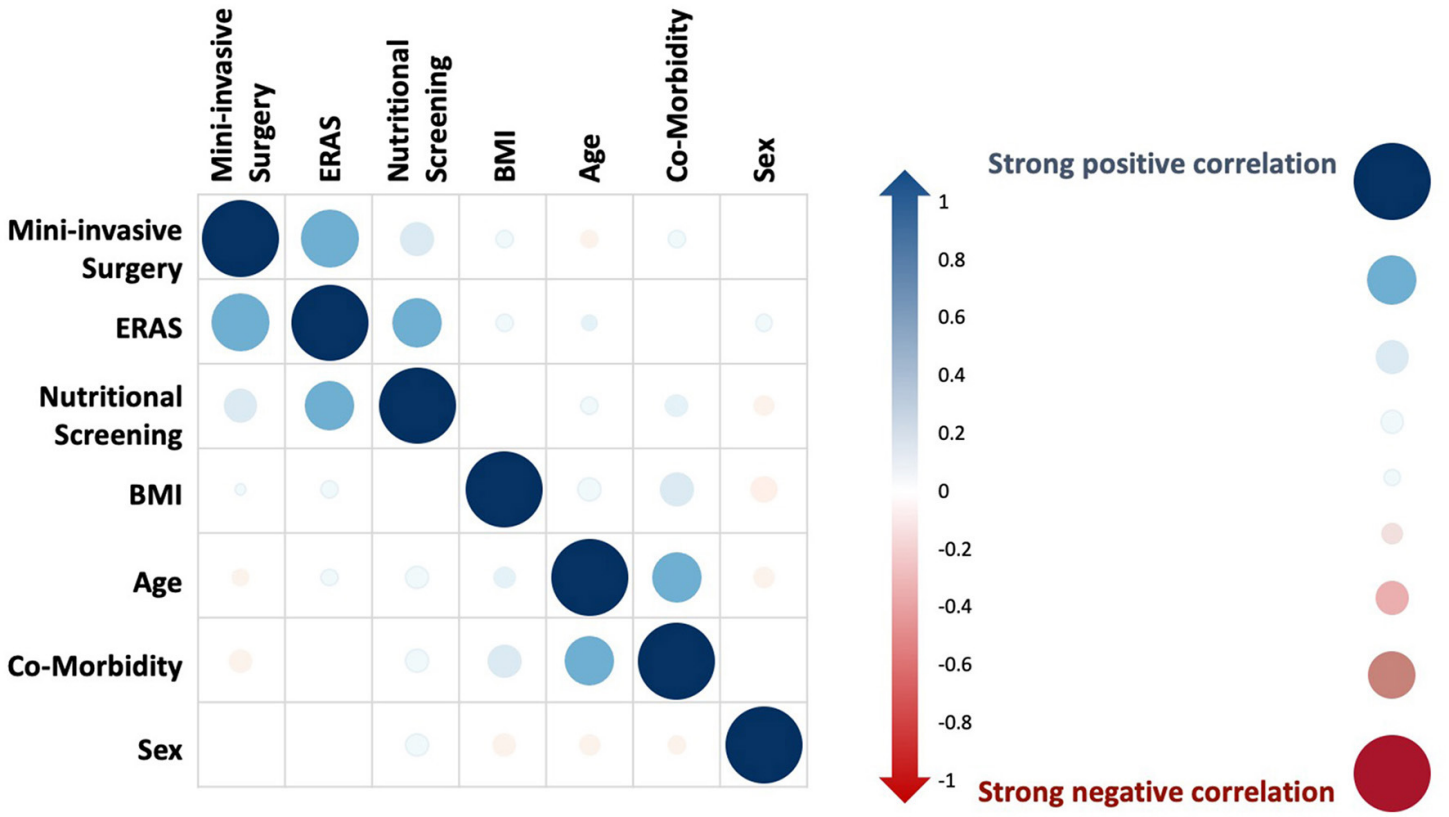
Partial correlations of endogenous variables. On the right side, the legend presents bubble dimensions and colors to express strong positive (blue) and negative (red) correlations among endogenous variables (mini-invasive surgery, ERAS, nutritional screening, BMI, age, comorbidity, and sex).

First, the variables were tested to evaluate possible correlations, and factor analyses (EFA and CFA) documented the consistency of the dataset, particularly with respect to sampling adequacy and variances (Supplementary Table 6). Then, the partial correlations were evaluated among clinical variables, and the results were consistent with the clinical assumptions (i.e., age correlated with comorbidity, ERAS practice correlated with MIS, and nutritional screening), as presented in Figure 2 and Table 2.

**Table 2:** Partial correlation of clinical variables^*^.

	**Age**	**Sex**	**Co-morbidity**	**BMI**	**Mini-invasive surgery**	**ERAS**	**Nutritional screening**
**Partial correlations**							
Age	1	−0.05	**0.43**	0.06	−0.03	0.04	0.06
Sex	−0.05	1	−0.03	−0.08	0	0.04	−0.07
Co-morbidity	**0.43**	−0.03	1	0.16	−0.04	−0.01	0.07
BMI	0.06	−0.08	0.16	1	0.02	0.03	0.02
Mini-invasive surgery	−0.03	0	−0.04	0.02	1	**0.41**	0.11
ERAS	0.04	0.04	−0.01	0.03	**0.41**	1	**0.30**
Nutritional screening	0.06	−0.07	0.07	0.02	0.11	**0.30**	1

* Bartlett's test of sphericity suggested a sufficient significant correlation in the data for factor analysis [Chisq (45) = 2475.27, p < 0.001), rejecting the null H0-hypothesis (absence of correlation). Bold is for statistical significance.

Factor analysis was conducted to test complexity and uniqueness. On this basis, a latent variable that could mirror patients' *performance status* based on unrelated independent (non-collinear) variables was computed. As presented in Table 3, nutritional screening and comorbidity were the endogenous variables displaying the lowest uniqueness level, thus the most appropriate candidates to be included in the computation of the latent variable.

**Table 3:** Complexity and uniqueness of variables.

**Variable**	**Complexity**	**Uniqueness**
ERAS	1.03	0.31
Tumor location	1.15	0.51
Mini-invasive surgery	1.09	0.64
Co-morbidity	1	4.31E-03
Age	1.05	0.81
Nutritional screening	1	4.73E-03
30-Days complications	1.02	0.45
LOS (Median)	1.91	0.53
Sex	2.08	0.91
BMI	1.49	0.91

LOS, Length of post-operative hospital stay; 30-Days Complications, 30-days post-operative complications. Bold is for statistical significance.

### SEM model and the value of nutritional screening

**Figure 3:**
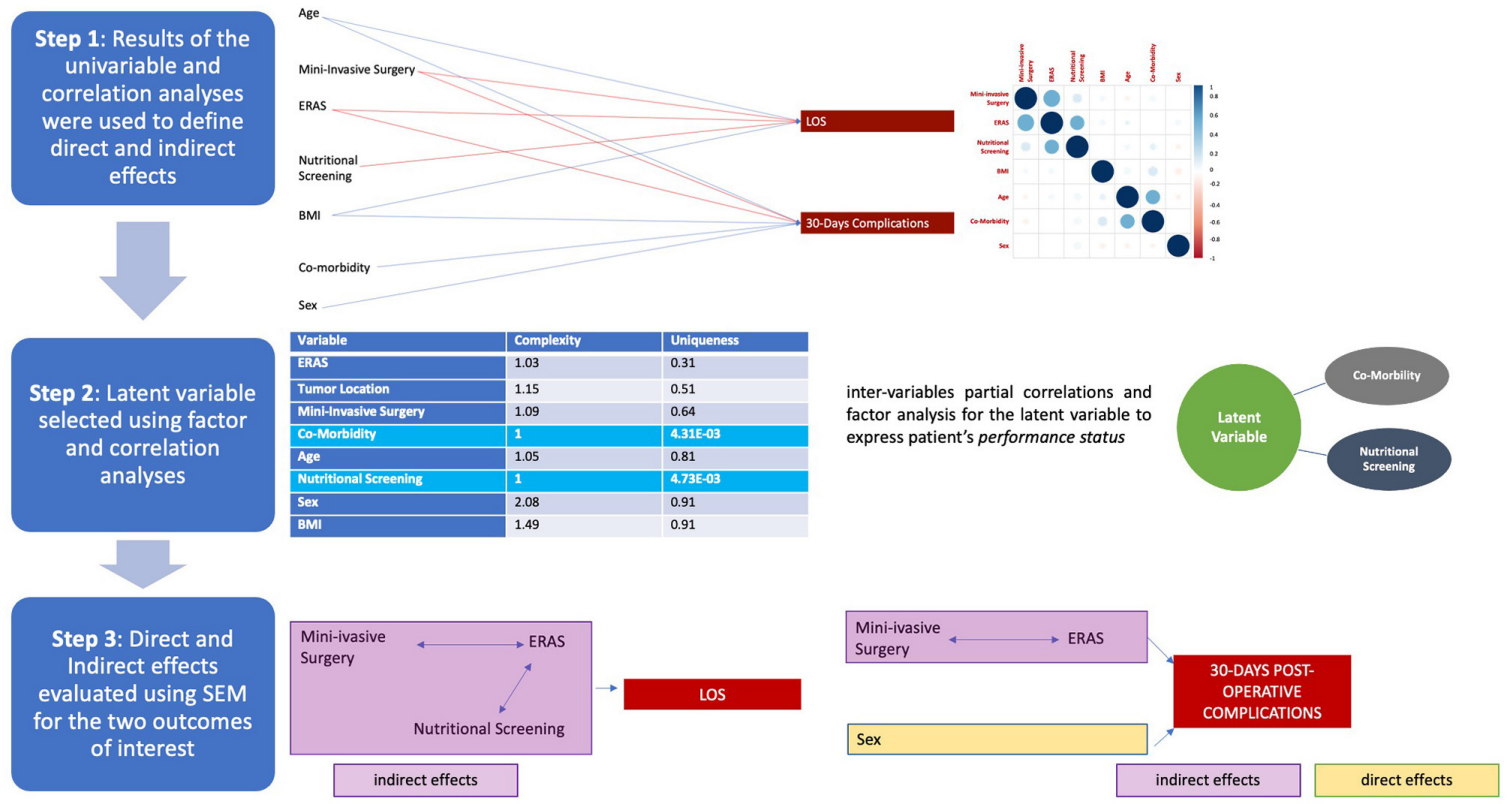
Steps for structural equation modeling (SEM) analysis: the process is described starting from the top as illustrated in the process diagram depicted on the left side of the picture. Steps are presented in the same sequential order as the statistical analyses. First step: univariable analyses and partial correlations. In the first part, the results of statistical value reported in Table 1 are depicted, with the positive (blue) or negative (red) correlation of variables with the outcomes [i.e., increased age correlated with increased length of hospital stay (LOS), blue line; whereas the use of mini-invasive surgery negatively correlated with the same outcome, red line]; the correlation diagram is the same as presented in Figure 2. Second step: definition of the latent variable according to factor analysis and based on those variables presenting greater uniqueness criteria: comorbidity and nutritional screening. Third step: finally, the final stage is depicted at the bottom of the figure (the definition of direct–yellow and indirect–pink effects on the outcomes).

These findings were computed using SEM and path analyses to evaluate the direct and indirect effects on the outcomes of interest, as presented in Figure 3. With this approach, it was documented that the latent variable/patients' *performance status* was best explained by the use of preoperative nutritional screening (*p* 0·004). Following, the correlations among clinical variables were shown, in particular: ERAS, tumor location; MIS, tumor location; co-morbidity, age; MIS, ERAS; nutritional screening, ERAS, *p* < 0·001.

**Figure 4:**
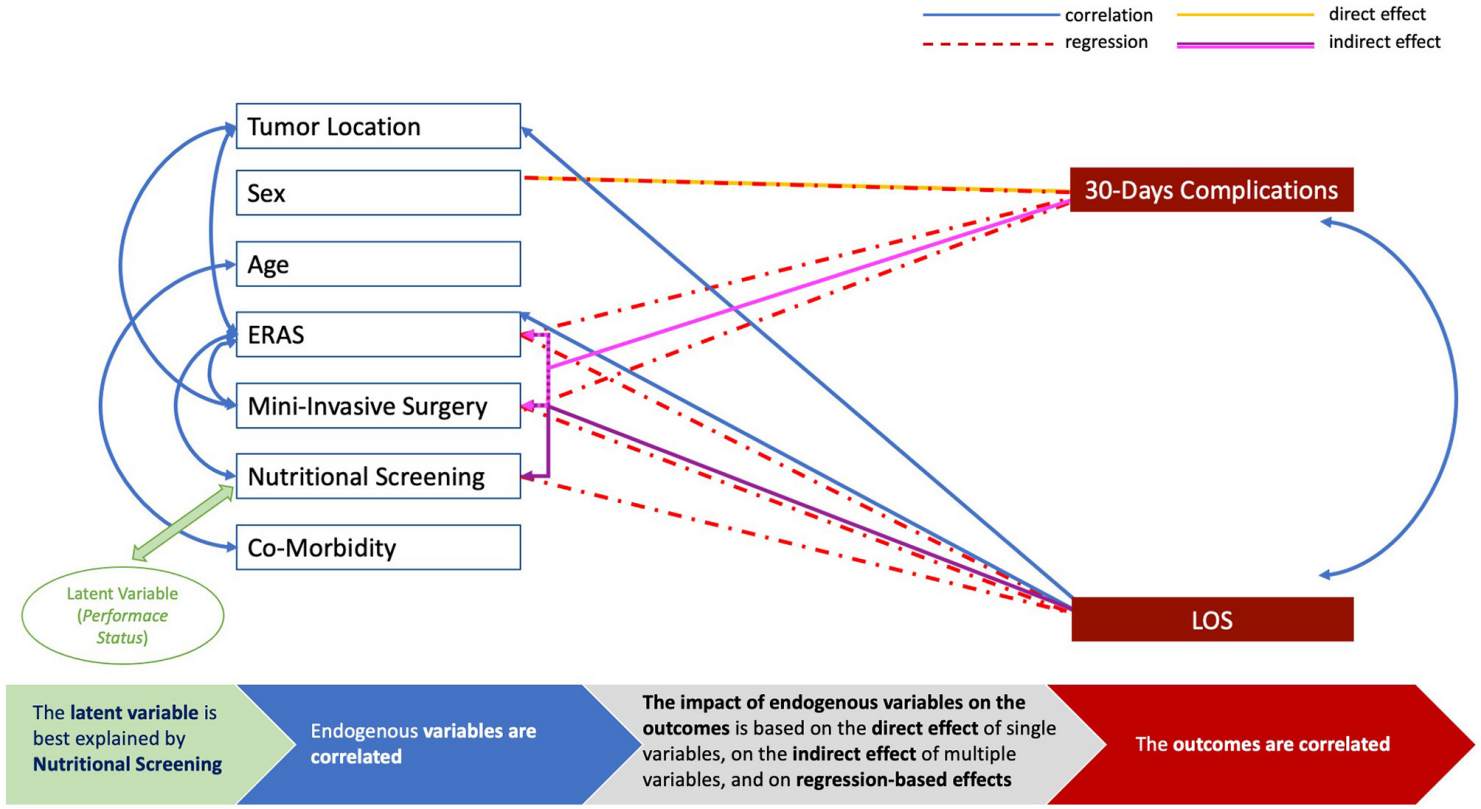
Graphical representation of the structural equation modeling (SEM) analysis. The blue boxes represent endogenous variables and the blue arrows are depicted to link inter-variables correlations of statistical value (*p* < 0·05, see Table 4); the red boxes are for the outcomes of interest (LOS, length of postoperative hospital stay; 30-day complications, 30-day postoperative complications); the green box is for the latent variable to represent patients' *performance status*, and the green arrow is to link this latent factor with the endogenous variable of statistical value (*p* < 0·05, see Table 4); the red arrows show regression-based effects with statistical significance (*p* < 0·05, see Table 4); the yellow arrows show direct effects of statistical value (*p* < 0·05, see Table 4); the purple arrows show the indirect effect with statistical significance (*p* < 0·05, see Table 4). The bottom line provides an explanation of the SEM analysis.

Finally, the impact of the clinical variables on the outcomes was disclosed, and based on direct effects (complications: sex, *p*0·001), indirect effects (LOS: MIS-ERAS-nutritional screening, *p* < 0·001; complications: MIS–ERAS, *p*0·001), and regression-based effects (LOS: ERAS *p* < 0·001, nutritional screening, *p*0·021; MIS, *p* < 0·001; complications, ERAS, and MIS, *p* < 0·001, sex, *p*0·001). As expected, LOS and complications were correlated (*p* < 0·001, Table 4). A graphical representation of the SEM analysis is presented in Figure 4 and all its parts are detailed in the Supplementary Video. In addition, the sum of the effects was significant (*p*0·003), and this model presented optimal performance (*p* < 0·001), as shown in Table 4.

**Table 4:** Structural equation modeling analysis^*^.

**Regression**	**Coefficient**	**SE**	**95% CI**	**z -test**	***p*-value**
**(A) LOS**					
Mini-invasive surgery	−0.06	0.003	[−0.066, −0.053]	−17.802	**0.000**
ERAS	−0.127	0.007	[−0.141, −0.113]	−17.551	**0.000**
Nutritional screening	−0.178	0.027	[−0.23, −0.124]	−6.61	**0.021**
BMI	−0.003	0.002	[−0.008. 0.002]	−1.055	0.291
Age	0	0.001	[−0.001, 0.002]	0.471	0.638
**(B) 30-Days complications**					
Age	0.00	0.001	[ −0.002, 0.002]	−0.142	0.887
BMI	0.00	0.003	[−0.005, 0.005]	0.021	0.983
ERAS	−0.092	0.026	[−0.143, −0.04]	−3.599	**0.000**
Sex	−0.065	0.02	[−0.103, −0.026]	−3.31	**0.001**
Mini-invasive surgery	−0.086	0.024	[−0.136, −0.041 ]	−3.57	**< 0.001**
Co-morbidity	0.047	0.026	[−0.003, 0.095]	1.824	0.068
**Latent variable**					
Co-morbidity	1	0	[1, 1]	NA	NA
Nutritional screening	0.091	0.032	[0.008, 0.137]	2.889	**0.004**
**Correlation**					
ERAS–tumor location	0.27	0.01	[0.25, 0.30]	22.36	**0.000**
Mini-invasive surgery–tumor location	0.21	0.01	[0.18, 0.23]	16.67	**0.000**
Co-morbidity–age	2.37	0.14	[2.09, 2.63]	16.52	**0.000**
LOS–ERAS	−0.04	0.005	[−0.056, −0.035]	−8.63	**0.000**
LOS–tumor location	−0.12	0.01	[ −0.15, −0.10]	−10.55	**0.000**
LOS–mini-invasive surgery	−0.05	0.005	[−0.06, −0.04]	−9.87	0.77
Co-morbidity–LOS	0.005	0.004	[−0.003, 0.014]	1.25	0.21
LOS−30-days complications	0.07	0.005	[0.06, 0.08]	14.55	**0.000**
Mini-invasive surgery–ERAS	0.11	0.005	[0.10, 0.12]	21.25	**0.000**
Nutritional screening–ERAS	0.03	0.004	[0.03, 0.04]	10.96	**0.000**
**Effects**					
Direct effect−30-days complications^§^	−0.06	0.02	[−0.10, −0.02]	−3.30	**0.001**
Indirect effect–LOS^§§^	−0.001	0.00	[−0.002, −0.001]	−5.45	**0.000**
Indirect effect−30-days complications^§§§^	0.008	0.002	[0.003, 0.01]	3.4	**0.001**
Total indirect effects	0.007	0.002	[0.002, 0.011]	2.81	**0.005**
Total effects	−0.05	0.02	[−0.09, −0.02]	−2.94	**0.003**

§ Direct Effect–30, Days Complications; Sex vs. 30-Days Complications.

§§ Indirect Effect–LOS: Mini-invasive Surgery–ERAS–Nutritional Screening vs. LOS.

§§§ Indirect Effect–30-Days Complications: Mini-invasive Surgery - ERAS vs. 30-Days Complications.

LOS, Length of post-operative hospital stay above the median value; 30-Days Complications, 30-days post-operative complications.

* Model features: Chi-square: χ^2^ 207.358 (P < 0.001); Root Mean Square Error of Approximation (RMSEA): 0.073 [95% CI (0.06, 0.08)]; Standardized Root Mean Square Residual (SRMR): 0.06; Comparative Fit Index (CFI): 0.0 (lowest)–1.0 (optimal): 0.923; Goodness-Fit Index (GFI): 0.0 (lowest) –1.0 (optimal): 0.97.

Bold is for statistical significance.

## Discussion

This study disclosed the inter-variable correlation among multiple features that are routinely evaluated in clinical studies in relation to adverse events, including patients' clinical factors (age, BMI, and comorbidities) and key elements of GI cancer care (invasiveness of the procedure, ERAS application, and preoperative nutritional screening).

It was demonstrated that these features are correlated, these correlations are consistent, and they produce direct and indirect effects on the outcomes: prolonged hospitalization and an increased rate of postoperative complications.

The results reported here are in line with the literature documenting that ERAS (22), MIS (23), and nutritional screening (24) are all beneficial in surgical oncology; however, the process used to support and document the findings is new in this field and disclosed a reliable path of relations among clinical features/variables.

In particular, the SEM analysis highlighted the decisive relationship between ERAS and MIS in reducing postoperative complications and the direct effect of several measures, including nutritional screening, in reducing LOS. Moreover, the latent factor we used to depict patients' *performance status* was best explained by the practice of screening patients for malnutrition, even if the focus was more on the practice itself than on the tool utilized, given the few discrepancies among the participating institutions. However, it is important to remark that this latent variable is just a closer approximation of the construct and not a real measurable condition.

It is also essential to stress the advantages of SEM over conventional multiple regression analyses for greater statistical power. Indeed, SEM is a similar but more powerful tool, since it evaluates linear causal relations among variables while simultaneously accounting for the measurement error (25).

The SEM analysis was developed to estimate factor analysis coefficients by the Swedish statistician–psychometrician Karl Jöreskog in the 70's and based on the linear structural relationship (LISREL) approach to address the issue of causality among variables, a fundamental topic in the field of econometrics and associated with the path analysis. The path analysis was indeed developed to part the correlation of variables into different segments in order to interpret their effects, assuming that some variables are related by a causal effect, and to estimate the magnitude of this correlation. Through these estimates, it was possible to provide information on the underlying random process and the technique provided the basis for structural equation models, later implemented and adopted by the R packages (26–29).

In the model herein presented, the total sum of effects (direct and indirect) was significant. One of the strengths of this study is indeed the methodology used. Although SEM was not conceived as causal modeling, these results revealed that the variables were significantly correlated and performed coherently with clinical assumptions. While largely used in psychology, SEM application in medical research has been highly advocated (30), but currently, it has been somewhat limited to psychiatry and epidemiology (31, 32).

However, this is surprising, as the contribution of SEM to clinical research is substantial and it is unique in this field. From a clinical point of view, it showed that the contribution of each item/variable is valuable and strong; however, given the inter-variable correlation, a multidisciplinary approach is the key when interpreting results. Indeed, the vast majority of multivariable analyses involuntarily ignore measurement error by not modeling it clearly, whereas SEM models estimate this variance for both independent and dependent variables (33).

With this in mind, the results highlighted by the present research help clinicians in understanding the strong contribution of both nutritional screening and mini-invasive surgery in the setting of enhanced recovery protocols in protecting patients from adverse outcomes (complications and prolonged hospital stay, also inter-correlated). Thus, the identification of the value of a single ERAS item over the others in several disciplines (34–36) may seem pleonastic.

On the other hand, when discussing study limitations, it should be noticed that the dataset analyzed was large and robust after excluding patients with missing data but included patients with different GI cancers, regardless of their stage. In relation to this issue, it should be also noted that several ERAS guidelines were published over the years (37), including those for CRC, UGI, and HPB surgeries, all based on the same principle of pre-habilitation, correction of deficits, and enhanced recovery of patients. In particular, there are few items in common among the different surgeries included in the present study, such as preoperative nutrition, peri-operative immuno/pharmaconutrition, nasogastric tube decompression, use of drains, postoperative artificial nutrition/intake, fluid management/balance, analgesic/anesthetic management (Supplementary Table 7).

Another set of possible discordant data lies in the relatively low rate of patients who received perioperative nutritional support (before and/or after surgery), in total less than one quarter, lower compared with previous experiences (38). For this reason and heterogeneous management (including oral immunonutrient supplementation, enteral feeding, and parenteral support/nutrition, often in combination) used in the setting of institutional protocols, or to correct malnutrition, or to support patients with complications, or even administered for all of these motivations, perioperative nutritional support was not included in the SEM analysis in relation to the outcomes of interest. However, when focusing on the subset of 358 patients homogeneously screened using MUST questionnaires, it could be noted that the rate of patients treated with nutritional support was more than double in those screened as MUST 2–5 compared with MUST 0 (25·0 *vs*. 9·3%), consistent with the appropriateness of the screening and treatment.

In addition, to counterbalance this issue, more than half of the entire cohort was treated in compliance with ERAS practice; thus, oral intake commenced within 24 h, following surgical resection.

With respect to other possible limitations, additional features such as ASA or other risk factor scores (i.e., P-Possum and others) were not collected. Nevertheless, patients' age was analyzed and correlated with the presence of comorbidities. Given the population analyzed (patients with cancer), a cutoff for comorbidity was set with a Charlson index >3, since patients with this value have a 10-year survival rate of 77·5% (39), greater than the mean survival rate of the GI cancers we analyzed. Similarly, previous studies identified seven core items with an adherence >80% (19), and this cutoff was adopted to define the ERAS protocol. Moreover, the analyses of this large dataset were focused on the common ground of ERAS, MIS, and nutritional screening, since the evaluation of all of the ERAS items and, to the same extent, the severity of postoperative complications were beyond the scope of this research but can be the object of future investigations.

## Conclusion

In this study, significant evidence for the benefits of MIS, ERAS, and nutritional screening in patients with cancer undergoing GI surgery was added, exploring the interrelation of variables and sustaining the multidisciplinary approach. Therefore, the exploration of this methodology in all branches of clinical research should be encouraged, particularly when evaluating outcomes in relation to patients' factors or practices that may have an impact on these features before an intervention.

## Data availability statement

The original contributions presented in the study are included in the article/Supplementary material, further inquiries can be directed to the corresponding author.

## Ethics statement

All procedures performed in studies involving human participants were in accordance with the ethical standards and with the 1964 Helsinki Declaration and its later amendments or comparable ethical standards. All patients provided written informed consent for the surgical procedures. This study was preliminary part of the Nutracare Project approved by the IRB at Fondazione Policlinico Universitario A. Gemelli, Rome. Written informed consent for participation was not required for this study in accordance with the national legislation and the institutional requirements.

## Author contributions

LL, RC, VC, GS, PD, MT, and DD'U: conceptualization, formal analysis, methodology, project administration, resources, and software. FI, MMe, CP, PP, AP, PF, AM, EF, AAv, FB, MN, RP, AB, FT, AAg, RF, VQ, MMi, DR, MRi, PD'E, MRh, and CC: data curation. LL, RC, VC, GS, PD, FI, MT, MMe, CP, PP, AP, PF, AM, EF, AAv, FB, MN, RP, AB, FT, AAg, RF, VQ, MMi, DR, MRi, PD'E, MRh, CC, and DD'U: investigation, writing, reviewing, and editing. LL, RC, VC, PD, FI, MT, MMe, CP, PP, AP, PF, EF, AAv, FB, MN, and DD'U: funding. PP, PF, PD, and DD'U: supervision and validation. LL, RC, VC, GS, PD, FI, MT, MMe, CP, PP, AP, PF, EF, AAv, FB, MN, and DD'U: roles/writing the original draft. All authors contributed to the article and approved the submitted version.

## Supplementary material

Supplementary Figure 1 **(A)** Institutional settings and volumes of surgeries for **(B)**. colorectal (CRC), **(C)** gastroesophageal (UGI), **(D)** hepatobiliary (HPB), and **(E)** peritoneal surface malignancies (PSM) in participating institutions each year (data presented as percentages).

Supplementary Figure 2 Institutional setting, clinical management, and nutritional screening at participating institutions.

Supplementary Table 1 Clinical features of the case series.

Supplementary Table 2 Clinical features according to cancer site in colorectal, gastroesophageal and hepatobiliary-pancreatic cancer patients.

Supplementary Table 3 Univariable analysis in colorectal cancer patients.

Supplementary Table 4 Univariable analysis in gastroesophageal cancer patients.

Supplementary Table 5 Univariable analysis in hepatobiliary-pancreatic cancer patients.

Supplementary Table 6 Correlations and variables.

Supplementary Table 7 ERAS guidelines synthesis.

Supplementary Video Video with a graphical representation of the structural equation modeling (SEM) analysis.
