# Supplementary figures and images for: The impact of preoperative nutritional screening, ERAS protocol, and mini-invasive surgery in surgical oncology: A multi-institutional SEM analysis of patients with digestive cancer
Source: Front Nutr. 2023 Mar 16;10:1041153. doi: 10.3389/fnut.2023.1041153 (PMC10063158; doi:10.3389/fnut.2023.1041153)

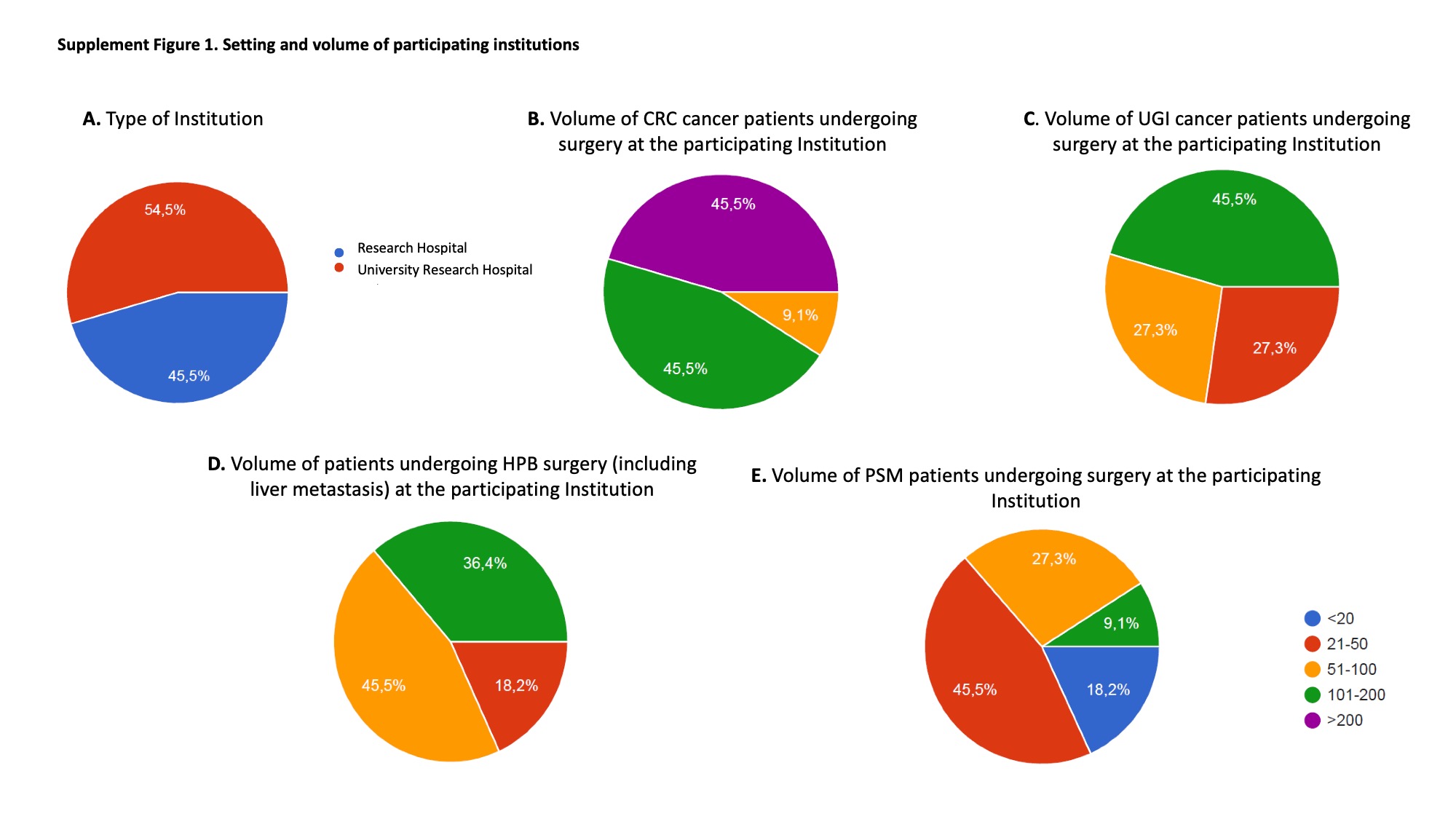

Supplement: Supplementary Figure 1 — (A) Institutional settings and volumes of surgeries for (B). colorectal (CRC), (C) gastroesophageal (UGI), (D) hepatobiliary (HPB), and (E) peritoneal surface malignancies (PSM) in participating institutions each year (data presented as percentages). [file Image_1.jpeg]

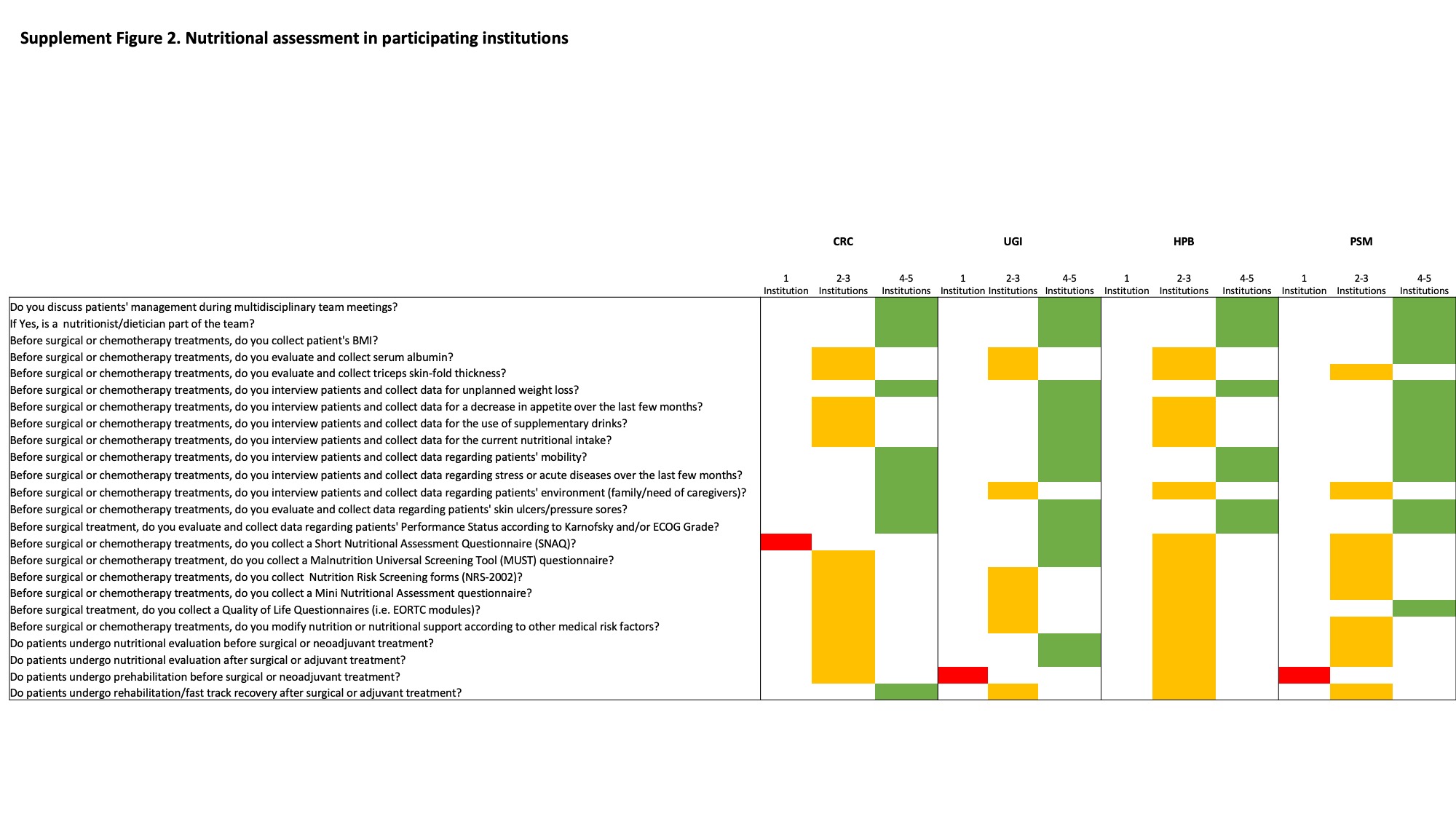

Supplement: Supplementary Figure 2 — Institutional setting, clinical management, and nutritional screening at participating institutions. [file Image_2.jpeg]
