# Supplementary material for: The impact of preoperative nutritional screening, ERAS protocol, and mini-invasive surgery in surgical oncology: A multi-institutional SEM analysis of patients with digestive cancer
Source: Front Nutr. 2023 Mar 16;10:1041153. doi: 10.3389/fnut.2023.1041153 (PMC10063158; doi:10.3389/fnut.2023.1041153)
Supplement: Supplementary Table 1 — Clinical features of the case series. [file Data_Sheet_1.docx]

**The impact of pre-operative nutritional screening, ERAS protocol and mini-invasive surgery in surgical oncology: a multi-institutional SEM analysis of digestive cancer patients**

Laura Lorenzon MD PhD^1^, Riccardo Caccialanza MD^2^, Valentina Casalone BSc^3^, Gloria Santoro PhD^1^, Paolo Delrio MD^4^, Francesco Izzo MD^4^, Marco Tonello MD PhD^5^, Maria Cristina Mele MD^1^, Carmelo Pozzo MD^1^, Paolo Pedrazzoli MD^2^, Andrea Pietrabissa MD^2^, Piero Fenu MD^3^, Alfredo Mellano MD^3^, Elisabetta Fenocchio MD^3^, Antonio Avallone MD^4^, Francesca Bergamo MD^5^, Maria Teresa Nardi MD^5^, Roberto Persiani MD^1^, Alberto Biondi MD^1^, Flavio Tirelli MD^1^, Annamaria Agnes MD^1^, Renato Ferraris MD^3^, Virginia Quarà MD^3^, Michela Milanesio MD^3^, Dario Ribero MD^3^, Marilena Rinaldi MD^3^, Paola D'Elia BSc^3^, Maurizio Rho MD^4^, Carola Cenzi PhD^5^, Domenico D'Ugo MD^1^

^1^Fondazione Policlinico Universitario Agostino Gemelli IRCCS, Catholic University, Rome, Italy

^2^Fondazione IRCCS Policlinico San Matteo, Pavia, Italy

^3^Candiolo Cancer Institute - IRCCS, Turin, Italy

^4^Istituto Nazionale per lo Studio e la Cura dei Tumori Fondazione Giovanni Pascale IRCCS, Naples, Italy

^5^Veneto Institute of Oncology IOV-IRCCS, Padua, Italy

**Corresponding author.** Laura Lorenzon, Fondazione Policlinico Universitario A. Gemelli, Catholic University, Largo Francesco Vito 1, 00168 Rome, Italy. email: [laura.lorenzon@policlinicogemelli.it](mailto:laura.lorenzon@policlinicogemelli.it); phone number: +39-0630154974; fax +39-0630158015 **ORCID ID** orcid.org/0000-0001-6736-0383; **Twitter** @LauraLorenzonMD

**Supplementary Materials - Index**

| **Setting evaluation - qualitative analysis** | *pag. 2* |
| --- | --- |
| **Results** |  |
| Supplementary Table 1 | *pag. 3* |
| Supplementary Table 2 | *pag. 4* |
| Supplementary Table 3 | *pag. 5* |
| Supplementary Table 4 | *pag. 6* |
| Supplementary Table 5 | *pag. 7* |
| **Structural equation modelling analysis** |  |
| Supplementary Table 6 | *pag. 8* |
| Model Features | *pag. 8* |
| **ERAS Guidelines Synthesis**  Supplementary Table 7 | *pag. 9* |

**Setting evaluation.** A qualitative analysis was conducted among surgeons from participating centers to describe: the institutional volumes for colorectal (CRC), gastroesophageal (UGI), hepatobiliary-pancreatic (HPB) and peritoneal surface malignancies (PSM) undergoing surgery each year; the presence of physician dedicated to clinical nutrition/dietician as part of the multidisciplinary team; use of ERAS protocols/fast track recovery and nutritional assessment before/after surgery, before/after neoadjuvant or adjuvant treatments; variables assessed at pre-admission/hospitalization in relation to the nutritional status of the patient; use of nutritional screening tools including the MUST, NRS-2002, MNA; evaluation of BMI, ASA, serum albumin, unplanned weight loss, decreased appetite, use of supplementation drinks, mobility, family environment, morbidity and patient therapy, performance status. Institutions were categorized as Research Hospitals or University Research Hospitals, whereas institutional volumes were categorized according to cancer type and as <20, 21-50, 51-100, 101-200, >200 resections/year.

As illustrated in Supplement Figure 1, almost the totality of the Institutions could be ranked as high-volume for CRC surgery according to the cut-off set by the Italian Ministry of Health (www.oncoguida.it). Also, the volume of UGI and HPB surgeries was high in the majority of the cases, and, as expected, it was medium-low for PSM, Supplement Figure 1.

The clinical practice in relation to nutritional screening, nutritional support and recovery pathway has been recorded and categorized on the basis of cancer type, to highlight items and variables consistently collected at each site. As shown, the key elements nutritional evaluations are routinely collected in each sub-specialty, however few discrepancies could be noted for HPB and CRC surgeries, Supplement Figure 2.

**Results**

**Supplementary Table 1. Clinical features of the case series**

|  | **Overall Population** |
| --- | --- |
| **Age (years)** |  |
| Mean; SD | 65.9; 12.3 |
| Median | 67.0 |
| Range | 19.0-96.0 |
| **Sex - n (%)** |  |
| M | 845 (51.3%) |
| F | 803 (48.7%) |
| M/F | 1.05 |
| Total | 1648 (100.0%) |
| **Tumor location - n (%)** |  |
| Colorectal | 1041 (63.2%) |
| Gastroesophageal | 177 (10.7%) |
| Hepatobiliary and pancreatic | 125 (7.6%) |
| Metastatic disease | 268 (16.3%) |
| Other | 37 (2.2%) |
| Total | 1648 (100.0%) |
| **Co-Morbidity - n (%)** |  |
| Yes | 1183 (71.8%) |
| No | 465 (28.2%) |
| Total | 1648 (100.0%) |
| **BMI** |  |
| Mean; SD | 25.8; 4.4 |
| Median | 25.4 |
| Range | 14.9-53.8 |
| **Mini-invasive surgery - n (%)** |  |
| Yes | 818 (49.6%) |
| No | 830 (50.4%) |
| Total | 1648 (100.0%) |
| **ERAS n (%)** |  |
| Yes (≥7 ERAS items applied) | 851 (51.6%) |
| No (<6 ERAS items applied) | 797 (48.4%) |
| Total | 1648 (100.0%) |
| **Nutritional Screening - n (%)** |  |
| Performed | 1409 (85.50%) |
| Not performed | 239 (14.50%) |
| Total | 1648 (100.0%) |
| **Lenght of post-operative hospital stay (days)** | |
| Mean; SD | 8.5; 12.3 |
| Median | 6.0 |
| Range | 1.0-367.0 |
| **30-days post-operative complications – n (%)** | |
| Yes | 440 (26.7%) |
| No | 1208 (73.3%) |
| Total | 1648 (100.0%) |

**Supplement Table 2. Clinical features according to cancer site in colorectal, gastroesophageal and hepatobiliary-pancreatic cancer patients**

|  | **CRC patients** | **UGI patients** | **HPB patients** |
| --- | --- | --- | --- |
| **Age (years)** |  |  |  |
| Mean; SD | 67.2; 12.1 | 68.1; 12.0 | 65.8; 10.5 |
| Median | 69.0 | 70.0 | 67.0 |
| Range | 19.0-91.0 | 34.0-96.0 | 20.0-85.0 |
| **Sex- n (%)** |  |  |  |
| M | 530 (50.9%) | 110 (62.2%) | 77 (61.6%) |
| F | 511 (49.1%) | 67 (37.8%) | 48 (38.4%) |
| M/F | 1.03 | 1.64 | 0.62 |
| **Co-Morbidity - n (%)** |  |  |  |
| Yes | 752 (72.2%) | 136 (76.8%) | 97 (77.6%) |
| No | 289 (27.8%) | 41 (23.2%) | 28 (22.4%) |
| Total | 1041(100.0%) | 177 (100.0%) | 125 (100.0%) |
| **BMI** |  |  |  |
| Mean; SD | 26.0; 4.3 | 24.9; 4.2 | 25.9; 3.9 |
| Median | 25.6 | 24.7 | 25.2 |
| Range | 15.6-53.8 | 14.9-38.6 | 18.5-40.1 |
| **Mini-invasive Surgery - n (%)** |  |  |  |
| Yes | 707 (67.9%) | 44 (24.9%) | 44 (35.2%) |
| No | 334 (32.1%) | 133 (75.1%) | 81 (64.8%) |
| Total | 1041(100.0%) | 177 (100.0%) | 125 (100.0%) |
| **ERAS- n (%)** |  |  |  |
| Yes (≥7 ERAS items applied) | 760 (73.0%) | 0 (0.0%) | 86 (68.8%) |
| No (<6 ERAS items applied) | 281 (27.0%) | 177 (100.0%) | 39 (31.2%) |
| Total | 1041(100.0%) | 177 (100.0%) | 125 (100.0%) |
| **Nutritional Screening – n(%)** |  |  |  |
| Yes | 962(92.4%) | 173 (97.7%) | 65 (52.0%) |
| No | 79 (7.6%) | 4 (2.3%) | 60 (48.0%) |
| Total | 1041(100.0%) | 177 (100.0%) | 125 (100.0%) |
| **Length of post-operative hospital stay (days)** | |  |  |
| Mean; SD | 6.6; 5.0 | 12.8; 11.4 | 10.7; 18.1 |
| Median | 5.00 | 10.00 | 7.00 |
| Range | 2.0–67.0 | 2.0-117.0 | 2.0-188.0 |
| **30-days post-operative complications – n (%)** | |  |  |
| Yes | 261 (25.1%) | 50 (28.2%) | 37 (29.6%) |
| No | 780 (74.9%) | 127 (71.8%) | 88 (70.4%) |
| Total | 1041(100.0%) | 177 (100.0%) | 125 (100.0%) |

**_CRC: colorectal; UGI: gastroesophageal, HPB: hepatobiliary-pancreatic_**

**Supplementary Table 3. Univariable analysis in colorectal cancer patients**

|  | **LOS**  **≤7 days** | **LOS**  **≥8 days** | **p- value** | **30-Days Complications**  **No** | **30-Days**  **Complications**  **Yes** | **p- value** |
| --- | --- | --- | --- | --- | --- | --- |
|  | **n patients (%)** | **n patients (%)** |  | **n patients (%)** | **n patients (%)** |  |
| **Mini-invasive Surgery** | |  |  |  |  |  |
| Yes | 594 (74.1%) | 113 (47.3%) | **<0.01^§^** | 570 (73.1%) | 137 (52.5%) | **<0.01^§^** |
| No | 208 (25.9%) | 126 (52.7%) |  | 210 (26.9%) | 124 (47.5%) |  |
| **Sex** |  |  |  |  |  |  |
| F | 412 (51.4%) | 99 (41.4%) | **<0.01^§^** | 399 (51.2%) | 112 (42.9%) | **0.02^§^** |
| M | 390 (48.6%) | 140 (58.6%) |  | 381 (48.8%) | 149 (57.1%) |  |
| **Nutritional Screening** | |  |  |  |  |  |
| Yes | 754 (94.0%) | 208 (87.0%) | **<0.01^§^** | 717 (91.9%) | 245 (93.9%) | 0.37^§^ |
| No | 48 (6.0%) | 31 (13.0%) |  | 63 (8.1%) | 16 (6.1%) |  |
| **Co-Morbidity** |  |  |  |  |  |  |
| Yes | 568 (70.8%) | 184 (77.0%) | 0.074^§^ | 544 (69.7%) | 208 (79.7%) | **<0.01^§^** |
| No | 234 (29.2%) | 55 (23.0%) |  | 236 (30.3%) | 53 (20.3%) |  |
| **ERAS (≥7 items applied)** | |  |  |  |  |  |
| Yes | 654 (81.6%) | 106 (44.4%) | **<0.01^§^** | 613 (78.6%) | 147 (56.3%) | **<0.01^§^** |
| No | 148 (18.4%) | 133 (55.6%) |  | 167 (21.4%) | 114 (43.7%) |  |
| **Age (years)** |  |  |  |  |  |  |
| Median (range) | 68.1 (19.0–89.0) | 70.0 (20.0–91.0) | **<0.01^§§§^** | 68.0 (18.8-89.0) | 70.9 (19.0-90.8) | **<0.01^§§§^** |
| **BMI** |  |  |  |  |  |  |
| Median (range) | 25.7 (16.0–54.0) | 25.4 (16.0–46.0) | **<0.01^§§§^** | 25.5 (15.5- 53.8) | 25.7 (15.6-46.2) | **<0.01^§§§^** |

**_LOS: Length of post-operative hospital stay above and below mean values; 30-Days Complications: 30-days post-operative complications_**

**_§ Chi square test; §§§ Mann-Whitney test._**

**Supplementary Table 4. Univariable analysis in gastroesophageal cancer patients**

|  | **LOS**  **≤13 days** | **LOS**  **≥14 days** | **p- value** | **30-Days Complications**  **No** | **30-Days**  **Complications**  **Yes** | **p- value** |
| --- | --- | --- | --- | --- | --- | --- |
|  | **n patients (%)** | **n patients (%)** |  | **n patients (%)** | **n patients (%)** |  |
| **Mini-invasive Surgery** | |  |  |  |  |  |
| Yes | 35 (27.8%) | 9 (17.7%) | 0.22^§^ | 34 (26.8%) | 10 (20.0%) | 0.45^§^ |
| No | 91 (72.2%) | 42 (82.3%) |  | 93 (73.2%) | 40 (80.0%) |  |
| **Sex** |  |  |  |  |  |  |
| F | 51 (40.5%) | 16 (31.4%) | 0.33^§^ | 55 (43.3%) | 12 (24.0%) | **0.02^§^** |
| M | 75 (59.5%) | 35 (68.6%) |  | 72 (56.7%) | 38 (76.0%) |  |
| **Nutritional Screening** | |  |  |  |  |  |
| Yes | 124 (98.4%) | 49 (96.1%) | 0.69^§^ | 125 (98.4%) | 48 (96.0%) | 0.67^§^ |
| No | 2 (1.6%) | 2 (3.9%) |  | 2 (1.6%) | 2 (4.0%) |  |
| **Co-Morbidity** |  |  |  |  |  |  |
| Yes | 93 (73.8%) | 43 (84.3%) | 0.19^§^ | 95 (74.8%) | 41 (82.0%) | 0.41^§^ |
| No | 33 (26.2%) | 8 (15.7%) |  | 32 (25.2%) | 9 (18.0%) |  |
| **Age (years)** |  |  |  |  |  |  |
| Median (range) | 71.0 (34.0–96.0) | 69.0 (43.0-92.0) | **<0.01^§§§^** | 70.0 (34.0-96.0) | 70.5 (41.0- 92.0) | **<0.01^§§§^** |
| **BMI** |  |  |  |  |  |  |
| Median (range) | 24.7 (15.0– 39.0) | 24.3 (16.0–35.0) | **<0.01^§§§^** | 24.7 (15.3-38.6) | 25.1 (14.9-35.1) | **<0.01^§§§^** |

**_LOS: Length of post-operative hospital stay above and below mean values; 30-Days Complications: 30-days post-operative complications_**

**_§ Chi square test; §§§ Mann-Whitney test._**

**Supplementary Table 5. Univariable analysis in hepatobiliary-pancreatic cancer patients**

|  | **LOS**  **≤11 days** | | **LOS**  **≥12 days** | | **p- value** | **30-Days Complications**  **No** | **30-Days**  **Complications**  **Yes** | **p- value** |
| --- | --- | --- | --- | --- | --- | --- | --- | --- |
|  | **n patients (%)** | | **n patients (%)** | |  | **n patients (%)** | **n patients (%)** |  |
| **Mini-invasive Surgery** | | | |  |  |  |  |  |
| Yes | | 41 (41.4%) | | 3 (11.5%) | **<0.01^§^** | 37 (42.1%) | 7 (18.9%) | **0.02^§^** |
| No | | 58 (58.6%) | | 23 (88.5%) |  | 51 (57.9%) | 30 (81.1%) |  |
| **Sex** | |  | |  |  |  |  |  |
| F | | 40 (40.4%) | | 8 (30.8%) | 0.50^§^ | 36 (40.9%) | 12 (32.4%) | 0.49^§^ |
| M | | 59 (59.6%) | | 18 (69.2%) |  | 52 (59.1%) | 25 (67.6%) |  |
| **Nutritional Screening** | | | |  |  |  |  |  |
| Yes | | 76 (76.8%) | | 10 (38.5%) | **<0.01^§^** | 59 (67.1%) | 27 (73.0%) | 0.65^§^ |
| No | | 23 (23.2%) | | 16 (61.5%) |  | 29 (32.9%) | 10 (27.0%) |  |
| **ERAS (≥7 items applied)** | |  | |  |  |  |  |  |
| Yes | | 61 (61.6%) | | 4 (15.4%) | **<0.01^§^** | 52 (59.1%) | 13 (35.1%) | **0.02^§^** |
| No | | 38 (38.4%) | | 22 (84.6%) |  | 36 (40.9%) | 24 (64.9%) |  |
| **Co-Morbidity** | | | |  |  |  |  |  |
| Yes | | 78 (78.8%) | | 19 (73.1%) | 0.72^§^ | 64 (72.7%) | 33 (89.2%) | 0.07^§^ |
| No | | 21 (21.2%) | | 7 (26.9%) |  | 24 (27.3%) | 4 (10.8%) |  |
| **Age (years)** | |  | |  |  |  |  |  |
| Median (range) | | 65.0 (20.0–83.0) | | 71.0 (51.0–85.0) | **<0.01**^§§§^ | 65.5 (20.0- 83.0) | 71.0 (51.0-85.0) | **<0.01^§§§^** |
| **BMI** | |  | |  |  |  |  |  |
| Median (range) | | 25.0 (19.0–40.0) | | 24.6 (18.0–32.0) | **<0.01**^§§§^ | 25.6 (19.9-40.1) | 24.5 (18.5-32.3) | **<0.01^§§§^** |

**_LOS: Length of post-operative hospital stay above and below mean values; 30-Days Complications: 30-days post-operative complications_**

**_§ Chi square test; §§§ Mann-Whitney test._**

**Structural equation modelling (SEM) analysis**

In brief, variables evaluated included: tumor location (qualitative), age (quantitative, normal distribution), sex (qualitative), co-morbidity (qualitative), BMI (quantitative, normal distribution), use of mini-invasive surgery (qualitative), ERAS (≥7 ERAS items applied: yes/no, qualitative), nutritional screening (qualitative), nutritional support (qualitative), 30-days post-operative complications (qualitative), LOS (quantitative, Poisson distribution).

The type of correlation was documented (Supplementary Table 6) to obtain the partial correlations as reported in Figure 2 and Table 2.

**Supplementary Table 6**

|  | **Age** | **BMI** | **Tumor Location** | **30-Days Complications** | **Co-Morbidity** | **Mini-Invasive Surgery** | **ERAS (≥7 items applied)** | **Nutritional Screening** | **Sex** | **LOS**  **_(median)_** |
| --- | --- | --- | --- | --- | --- | --- | --- | --- | --- | --- |
| **Age** | 1 | Pearson | Polyserial | Polyserial | Polyserial | Polyserial | Polyserial | Polyserial | Polyserial | Polyserial |
| **BMI** | 0.06526 | 1 | Polyserial | Polyserial | Polyserial | Polyserial | Polyserial | Polyserial | Polyserial | Polyserial |
| **Tumor Location** | 0.06824 | 0.1252 | 1 | Polychoric | Polychoric | Polychoric | Polychoric | Polychoric | Polychoric | Polychoric |
| **30-Days Complications** | 0.02458 | 0.006022 | -0.05034 | 1 | Polychoric | Polychoric | Polychoric | Polychoric | Polychoric | Polychoric |
| **Co-Morbidity** | 0.5766 | 0.2106 | -0.04581 | 0.1009 | 1 | Polychoric | Polychoric | Polychoric | Polychoric | Polychoric |
| **Mini-Invasive Surgery** | -0.0257 | 0.04794 | 0.4421 | -0.2548 | -0.08182 | 1 | Polychoric | Polychoric | Polychoric | Polychoric |
| **ERAS (≥7 items applied)** | 0.0519 | 0.06303 | 0.6138 | -0.2621 | -0.03101 | 0.704 | 1 | Polychoric | Polychoric | Polychoric |
| **Nutritional Screening** | 0.098 | 0.053 | 0.3155 | 0.08164 | 0.1402 | 0.3327 | 0.6708 | 1 | Polychoric | Polychoric |
| **Sex** | -0.06851 | -0.09668 | 0.02562 | -0.1498 | -0.0649 | 0.01306 | 0.0776 | -0.1369 | 1 | Polychoric |
| **LOS**  **_(median)_** | -0.01464 | -0.06943 | -0.4166 | 0.6078 | 0.05183 | -0.5329 | -0.5887 | -0.3965 | -0.03516 | 1 |

**_LOS: Length of post-operative hospital stay; 30-Days Complications: 30-days post-operative complications_**

The Kaiser, Meyer, Olkin (KMO) measure of sampling adequacy documented that data were appropriate for factor analysis (KMO = 0.68).

Finally, SEM analysis was performed to evaluate the impact of the endogenous variables on the outcomes. SEM included regressions (based on results of the univariable analysis), inter-variables correlations (based on results of the partial correlations), and the measure of direct and indirect effects (based both on univariable and partial correlation analyses). The model was chosen based on the following computations:

- Chi-square: χ^2^ 207.358 (P <0.001)
- ROOT MEAN SQUARE ERROR OF APPROXIMATION (RMSEA): 0.073 (95% CI [0.06, 0.08])
- STANDARDIZED ROOT MEAN SQUARE RESIDUAL (SRMR): 0.06
- COMPARATIVE FIT INDEX (CFI): 0.0 (lowest) - 1.0 (optimal): 0.923
- GOODNESS-FIT INDEX (GFI): 0.0 (lowest) - 1.0 (optimal): 0.97

The results evidenced the appropriateness of the model.

**Supplementary Table 7. ERAS Guidelines Synthesis**

| Liver Surgery | Colorectal | Pancreatic | Gastrectomy | Cytoreductive surgery | Esophagectomy |
| --- | --- | --- | --- | --- | --- |
| Joliat, GR., Kobayashi, K., Hasegawa, K. *et al.* Guidelines for Perioperative Care for Liver Surgery: Enhanced Recovery After Surgery (ERAS) Society Recommendations 2022. *World J Surg* 47, 11–34 (2023). | Gustafsson, U.O., Scott, M.J., Hubner, M. *et al.* Guidelines for Perioperative Care in Elective Colorectal Surgery: Enhanced Recovery After Surgery (ERAS^®^) Society Recommendations: 2018. *World J Surg* **43**, 659–695 (2019). | Melloul, E., Lassen, K., Roulin, D. *et al.* Guidelines for Perioperative Care for Pancreatoduodenectomy: Enhanced Recovery After Surgery (ERAS) Recommendations 2019. *World J Surg* **44**, 2056–2084 (2020). | Mortensen K, Nilsson M, Slim K, Schäfer M, Mariette C, Braga M, Carli F, Demartines N, Griffin SM, Lassen K; Enhanced Recovery After Surgery (ERAS®) Group. Consensus guidelines for enhanced recovery after gastrectomy: Enhanced Recovery After Surgery (ERAS®) Society recommendations. Br J Surg. 2014 Sep;101(10):1209-29. | Hübner M, Kusamura S, Villeneuve L, Al-Niaimi A, Alyami M, Balonov K, Bell J, Bristow R, Guiral DC, Fagotti A, Falcão LFR, Glehen O, Lambert L, Mack L, Muenster T, Piso P, Pocard M, Rau B, Sgarbura O, Somashekhar SP, Wadhwa A, Altman A, Fawcett W, Veerapong J, Nelson G. Guidelines for Perioperative Care in Cytoreductive Surgery (CRS) with or without hyperthermic IntraPEritoneal chemotherapy (HIPEC): Enhanced recovery after surgery (ERAS®) Society Recommendations - Part I: Preoperative and intraoperative management. Eur J Surg Oncol. 2020 Dec;46(12):2292-2310.  Hübner M, Kusamura S, Villeneuve L, Al-Niaimi A, Alyami M, Balonov K, Bell J, Bristow R, Guiral DC, Fagotti A, Falcão LFR, Glehen O, Lambert L, Mack L, Muenster T, Piso P, Pocard M, Rau B, Sgarbura O, Somashekhar SP, Wadhwa A, Altman A, Fawcett W, Veerapong J, Nelson G. Guidelines for Perioperative Care in Cytoreductive Surgery (CRS) with or without hyperthermic IntraPEritoneal chemotherapy (HIPEC): Enhanced Recovery After Surgery (ERAS®) Society Recommendations - Part II: Postoperative management and special considerations. Eur J Surg Oncol. 2020 Dec;46(12):2311-2323. | Low DE, Allum W, De Manzoni G, Ferri L, Immanuel A, Kuppusamy M, Law S, Lindblad M, Maynard N, Neal J, Pramesh CS, Scott M, Mark Smithers B, Addor V, Ljungqvist O. Guidelines for Perioperative Care in Esophagectomy: Enhanced Recovery After Surgery (ERAS^®^) Society Recommendations. World J Surg. 2019 Feb;43(2):299-330. doi: 10.1007/s00268-018-4786-4. PMID: 30276441. |
| 1. Preoperative counseling | 1. Preadmission information, education and counselling | 1. Preoperative counseling | 1. Preoperative nutrition | 1. Preadmission information, education and counselling | 1. Preoperative nutritional assessment and treatment |
| 2. Prehabilitation | 2. Preoperative optimisation | 2. Prehabilitation | 2. Preoperative oral pharmaconutrition | 2. Preoperative optimisation: alcohol, smoking, anemia | 2. Preoperative nutritional intervention |
| 3. Preoperative biliary drainage | 3. Prehabilitation | 3. Preoperative biliary drainage | 3. Access | 3. Physical exercise/prehabilitation | 3. Preoperative oral pharmaconutrition |
| 4. Preoperative smoking and alcohol cessation | 4. Preoperative nutritional care | 4. Preoperative smoking and alcohol consumption | 4. Wound catheters and TAP block | 4. Nutritional care: Screening, supplementation (oral, enteral, parenteral), immunonutrition | 4. Multidisciplinary tumor board |
| 5. Preoperative nutrition | 5. Management of Anaemia | 5. Preoperative nutrition | 5. Nasogastric/nasojejunal decompression | 5. Preoperative anaesthetic assessment | 5. Prehabilitation programs |
| 6. Perioperative oral immunonutrition | 6. Prevention of nausea and vomiting (PONV) | 6. Perioperative oral immunonutrition | 6. Perianastomotic drains | 6. Post-Operative Nausea and Vomiting (PONV) | 6. Timing of surgery following neoadjuvant therapy |
| 7. Preoperative fasting and preoperative carbohydrate load | 7. Pre-anaesthetic medication | 7. Preoperative fasting and treatment with carbohydrates | 7. Early postoperative diet and artificial nutrition | 7. Pre-anaesthetic medication | 7. Access |
| 8. Pre-anesthetic medication | 8. Antimicrobial prophylaxis and skin preparation | 8. Pre-anesthetic medication | 8. Audit | 8. Preoperative bowel preparation | 8. Choice of conduit |
| 9. Anti-thrombotic prophylaxis | 9. Bowel Preparation The same recommendation grade and quality of evidence | 9. Anti-thrombotic prophylaxis | 9. Preoperative counselling | 9. Preoperative fasting and carbohydrate treatment | 9. Role of pyloroplasty |
| 10. Preoperative steroids administration | 10. Preoperative fluid and electrolyte therapy | 10. Antimicrobial prophylaxis and skin preparation | 10. Preoperative smoking and alcohol consumption | 10. Antimicrobial prophylaxis and skin preparation | 10. Lymphadenectomy |
| 11. Antimicrobial prophylaxis and skin preparation | 11. Preoperative fasting and carbohydrate loading | 11. Epidural analgesia | 11. Oral bowel preparation | 11. Standard anaesthetic protocol | 11. Perianastomotic drains |
| 12. Minimally invasive surgery | 12. Standard Anaesthetic Protocol | 12. Postoperative intravenous and per oral analgesia | 12. Preoperative fasting and preoperative treatment with carbohydrates | 12. Intraoperative normothermia | 12. NG tube/gastric decompression |
| 13. Epidural, postoperative intravenous, and postoperative per oral analgesia | 13. Intraoperative fluid and electrolyte therapy | 13. Wound catheter and transversus abdominis plane (TAP) block | 13. Preanaesthetic medication | 13. Intraoperative normoglycemia | 13. Chest drain management following esophagectomy |
| 14. Wound catheter and transversus abdominis plane (TAP) block | 14. Preventing intraoperative hypothermia | 14. Postoperative nausea and vomiting (PONV) prophylaxis | 14. Antithrombotic prophylaxis | 14. Perioperative fluid management | 14. Routine use of enteric feeding tubes |
| 15. Prophylactic nasogastric intubation | 15. Surgical access | 15. Avoiding hypothermia | 15. Antimicrobial prophylaxis and skin preparation | 15. Transfusion and management of coagulopathy | 15. Esophagectomy: perioperative fluid management |
| 16. Prophylactic abdominal drainage | 16. Drainage of the peritoneal cavity and pelvis | 16. Postoperative glycemic control | 16. Epidural analgesia | 16. Abdominal and thoracic drains | 16. Anesthetic management |
| 17. Preventing intraoperative hypothermia | 17. Nasogastric Intubation | 17. Nasogastric intubation | 17. Intravenous analgesia | 17. Early extubation | 17. Two-lung ventilation |
| 18. Postoperative artificial nutrition and early oral intake | 18. Postoperative analgesia | 18. Fluid balance | 18. Anaesthetic management | 18. Nasogastric drainage | 18. One-lung ventilation |
| 19. Postoperative glycemic control | 19. Thromboprophylaxis Mechanical thromboprophylaxis | 19. Perianastomotic drainage | 19. PONV | 19. Urinary indwelling catheter | 19. Intensive care unit utilization |
| 20. Prevention of delayed gastric emptying (DGE) | 20. Postoperative fluid and electrolyte therapy | 20. Somatostatin analogues | 20. Avoiding hypothermia | 20. Prevention of postoperative ileus (including use of postoperative laxatives) |  |
| 21. Stimulation of bowel movement | 21. Urinary | 21. Urinary drainage | 21. Postoperative glycaemic control | 21. Postoperative analgesia |  |
| 22. Early and scheduled mobilization | 22. Prevention of postoperative ileus | 22. Delayed gastric emptying | 22. Fluid balance | 22. Perioperative nutritional care |  |
| 23. Postoperative nausea and vomiting (PONV) prophylaxis | 23. Postoperative glycaemic control | 23. Stimulation of bowel movement | 23. Urinary drainage | 23. Postoperative control of glucose |  |
| 24. Fluid management | 24. Postoperative nutritional care | 24. Postoperative artificial nutrition | 24. Stimulation of bowel movement | 24. Prophylaxis against thromboembolism |  |
| 25. Monitoring/Audit |  | 25. Early and scheduled mobilization | 25. Early and scheduled mobilization | 25. Prevention, early detection and treatment of HIPEC complications |  |
|  |  | 26. Minimal invasive surgery |  | 26. Early Mobilisation |  |
|  |  | 27. Audit |  | 27. Post-discharge care after CRS/HIPEC |  |
|  |  |  |  | 28. ERAS Audit and Reporting |  |
